# Experimental determination of TRIP-parameter K for mild- and high-strength low-alloy steels and a super martensitic filler material

**DOI:** 10.1186/s40064-016-2474-0

**Published:** 2016-06-17

**Authors:** Sebastian Neubert, Andreas Pittner, Michael Rethmeier

**Affiliations:** Department 9.3 - Component Safety/Welding Technology, BAM - Federal Institute for Materials Research and Testing, Unter den Eichen 87, 12205 Berlin, Germany

**Keywords:** Transformation induced plasticity (TRIP), Phase transformation, High-strength low-alloy steel, Super martensitic filler material, Thermo physical simulation, *Gleeble* experiments

## Abstract

A combined experimental numerical approach is applied to determine the transformation induced plasticity (TRIP)-parameter K for different strength low-alloy steels of grade S355J2+N and S960QL as well as the super martensitic filler CN13-4-IG containing 13 wt% chromium and 4 wt% nickel. The thermo-physical analyses were conducted using a *Gleeble*^®^ 3500 facility. The thermal histories of the specimens to be tested were extracted from corresponding simulations of a real gas metal arc weldment. In contrast to common TRIP-experiments which are based on complex specimens a simple flat specimen was utilized together with an engineering evaluation method. The evaluation method was validated with literature values for the TRIP-parameter. It could be shown that the proposed approach enables a correct description of the TRIP behavior.

## Background

Due to the global goal to reduce the CO_2_ emissions product development is governed by criterions to minimize the overall energy and resource consumption. With respect to the material design, high-strength low-alloy steels still enable the best compromise between light-weight constructions and processability. However, superior mechanical behavior is realized by special thermo-mechanical treatment. Consequently, thermal fabrication processes such as welding and cutting, have to take the heat sensitivity of these materials into account. In the case of fusion welding, the high level of yield strength cause higher residual stresses compared to conventional steel grades.

The knowledge about the heat effects of welding is the basis for an optimal design of components made of high-strength steels. In this context structural thermo-mechanical welding simulation is a tool that offers the detailed analyses of the evolution of the temperature field as well as the resulting thermo-mechanical response of the structure. Regarding a good description of engineering weld applications more and more complex models for welding simulation are necessary (Lindgren 2001). A bottle neck of welding simulation is the availability of temperature dependent material data (Lindgren 2001). Whereas the thermo-physical values like heat conductivity, density and specific heat do not differ much between alloys of a similar chemical composition. But the thermo-mechanical properties like stress–strain curves and transformation behavior are strongly dependent of the fabrication process of the steel. Regarding the transformation behavior the onset and finish temperature of solid state transformation will heavily influence the qualitative and quantitative evolution of residual stress for a welded specimen (Liu et al. 2008). Furthermore, the transformation induced plasticity (TRIP), a concomitant phenomenon of the solid state transformation, must be considered (Bhadeshia et al. 2007).

However, the experimental investigation of the material properties is very time consuming and costly. Thus, the present study focuses on an efficient engineering approach to evaluate the transformation behavior under consideration of the transformation induced plasticity. For this purpose, dilatometer experiments using the *Gleeble*^®^ 3500 facility have been performed. Three transformable metallic materials which differ in strength or alloy content were taken into account. Besides the high-strength steel S960QL (yield strength of 960 MPa) the structural mild steel S355J2+N (yield strength of 355 MPa) were chosen as low-alloyed material. To verify the applicability of the experimental method to determine the transformation induced plasticity a third material with high-alloy content will be tested. Doing this, weld metal test specimens of the super martensitic filler material CN13-4-IG (yield strength of 970 MPa) containing 13 wt% chromium and 4 wt% nickel were also evaluated.

In contrast to the conventional approach the dilatometer experiments are done without using complex and expensive cylindrical hollow specimens but with simple flat specimens that are easy to fabricate concerning costs and time. The applied load in terms of thermal cycles was extracted from numerical simulations of the temperature field on basis of thermo-couple measurements that recorded the temperature history for the GMA-weldments. During the execution of the dilatometer experiments an additional external tension and compression load below the yield point of the softer austenitic phase was applied (Table 2). After cooling down to room temperature the mechanical loads were released and the remaining plastic strain is caused by transformation induced plasticity. Based on the different stresses for the tensile and compression load case the TRIP-parameter K can be evaluated.

The experimental approach is validated by comparing the values for the TRIP-parameter K for the metal specimens with corresponding literature results (Franz et al. 2004; Ossenbrink 2009; Kasuya et al. 2014). Furthermore, this work presents values for the super martensitic filler material CN13-4-IG which have not been published yet.

### Theoretical background

The physical phenomenon transformation induced plasticity (TRIP) was already observed in 1919 by *Tiemann* (Tiemann 1919) and designated by Wassermann (1937). Here TRIP concerns solid phase transformable steels during (γ → α)-phase transformation when plastic yielding occurs although the level of existing load stresses are below the yield criterion of the involved softer solid phase (austenite). Case-specific mathematical models describing the TRIP-effect were derived by Anderson and Bishop (1962), Greenwood and Johnson (1965), Magee (and Paxton 1966), Abrassart (1972) and Leblond et al. (1989). A comprehensive summary can be found in Mitter (1987).

The following sections handle the experimental determination of TRIP-parameter K on steels with solid phase transformation behavior. If steel undergoes thermals cycles imposed by welding process, the total strain *ɛ* is composed by elastic $\varepsilon_{\text{el}}$, plastic $\varepsilon_{\text{pl}}$, thermal $\varepsilon_{\text{th}}$, transformation $\varepsilon_{\text{tr}}$ and transformation induced plasticity strain $\varepsilon_{\text{tp}}$, which is shown by Eq. 1 (Besserdich 1993):

1 $$\varepsilon = \varepsilon_{\text{el}} + \varepsilon_{\text{pl}} + \varepsilon_{\text{th}} + \varepsilon_{\text{tr}} + \varepsilon_{\text{tp}}$$

Assuming a constant uniaxial macro stress *σ* with K—material dependent transformation induced plasticity parameter and *f*(*w*)—function for describing present progress of transformation the TRIP-strain $\varepsilon_{\text{tp}}$ can be written in the following form (Besserdich et al. 1994):

2 $$\varepsilon_{\text{tp}} = Kf\left( w \right)\sigma$$

After the (*γ* → *α*)-phase transformation is completed, that means *f*(*w*) = 1, Eq. 2 gives the linear relationship:

3 $$\varepsilon_{\text{tp}} = K\sigma$$

Applying several different but during (γ → α)-phase transformation constant tensile or compressive loads cause an uniaxial macro stress *σ*, which must not exceed the yield point of the involved austenitic phase. Relieving these loads after (γ → α)-phase transformation is finished, the TRIP-strain $\varepsilon_{\text{tp}}$ will remain and can be evaluated at room temperature. Knowing *σ* and $\varepsilon_{\text{tp}}$ the TRIP-parameter K can be derived by Eq. 4:

4 $$K = \left. {{\text{d}}\varepsilon_{\text{tp}} (\sigma )/{\text{d}}\sigma } \right|_{\sigma \to 0}$$

### Experimental determination

With regards to the experimental test methodology the TRIP-parameter K was determined by Franz et al. (2004), Ossenbrink (2009), Kasuya et al. (2014), Besserdich et al. (1994), Ahrens (2003), Trapp (2010), Dalgic et al. (2008), Taleb et al. (2001), Besserdich et al. (2000). The TRIP-parameter K depends on many factors, e.g. the involved type of (γ → α)-phase transformation (during continuous cooling or isothermal conditions), the temperature, the load case (tensile or compressive) and internal inertial strains. Furthermore, the TRIP experiments are governed by the geometry of the test specimen which is discussed in literature controversially. For avoiding radial temperature gradients within the test specimen some authors (Besserdich et al. 1994; Ahrens 2003; Trapp 2010; Dalgic et al. 2008; Taleb et al. 2001; Besserdich et al. 2000) applied complex and thus costly hollow cylindrical specimen geometries. Instead, other scientists (Franz et al. 2004; Ossenbrink 2009; Kasuya et al. 2014; Dalgic et al. 2008) used round solid specimens. A sensitivity analysis, e.g. which sample geometry for the determination of TRIP-parameter *K* is more appropriate, cannot be found. The thermal conditions for austenitization are important and addressed in Franz et al. (2004), Kasuya et al. (2014), Besserdich et al. (1994), Ahrens (2003), Trapp (2010), Dalgic et al. (2008), Besserdich et al. (2000) where the specimens were heated up from room temperature to a maximum temperature range of 800–910 °C within several minutes. Subsequently, in order to ensure complete austenitization, the specimens were held at these temperatures for 5–30 min. After austenitization the specimens were cooled down to room temperature, whereby the applied thermal cycle is based on the transformation behavior of the considered steel specimen and the required condition of the resulting α-phase. In contrast, some authors (Ossenbrink 2009; Kasuya et al. 2014) used temperature cycles in accordance to thermo couple measurements of real welding experiments with peak temperatures of about 1100 °C. Thus, additionally grain growth and precipitation can be prevented.

In this research TRIP-experiments were performed to build up a material data base for numerical welding simulation. Therefore, based on executed GMA welding experiments, the thermal loads for the TRIP-experiments were extracted from a simulated welding temperature field that is validated against thermo couple measurements. The underlying method of thermal validation is exemplarily explained in Neubert et al. (2016). Because of that the tested high-strength steel specimens undergo the same thermo-physical and thermo-mechanical characteristics like in the real welding experiment. Furthermore, the flat specimen geometry results in a significant reduction of effort to fabricate the samples yielding in fewer restrictions regarding the quantity of performed dilatometer experiments.

## Design of TRIP-experiments

An important aspect is that the TRIP-experiments were performed by applying thermal cycles that correspond to the thermal history of real GMA welding experiments. Here, the pulsed GMA welding processes were done on steel sheets of S355J2+N and S960QL with matching and super martensitic filler materials by the same quantities as 55 cm/min welding speed, 0.74 kJ/mm energy input per unit length and a wiring feed rate with 9 m/min. The specimens were made of S355J2+N and S960QL steel sheets from the same heat as the plates used for the GMA welding experiments. The specimens for testing the super martensitic filler material CN13-4-IG were machined in transversal direction to the weld seam of a multi-pass weld metal test specimen (DIN EN ISO 15792-1). The averaged chemical composition of the multi-pass welded filler material was determined by electron probe micro analysis (EPMA). In this case a line scan of the cross-section using wavelength-dispersive X-ray spectroscopy gives 0.02 wt% carbon, 11.2 wt% chromium and 4.1 wt% nickel. The chemical compositions of the examined materials are given by Table 1.

**Table 1:** Chemical composition of tested materials in wt%

C	P	S	N	Cu	Nb	Ti	V	Al	Si	Mn	Cr	Mo	Ni
*Base material S355J2+N*													
0.16	0.012	0.008	0.01	0.06	0.005	0.002	0.001	0.045	0.028	1.47	0.057	0.01	0.028
*Base material S960QL*													
0.14	0.009	0.001	0.01	0.03	0.013	0.002	0.046	0.03	0.3	0.87	0.49	0.53	0.52
*Filler material CN13-4-IG*													
0.09	0.019	0.006	0.129	0.03	0.008	0.001	0.048	0.016	0.53	0.65	11.2	0.38	4.1

**Fig. 1:**
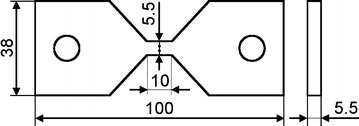
Flat specimen geometry. Dimensions of flat specimen geometry for materials S355J2+N, S960QL and CN13-4-IG. Measuring range is located at the middle of the flat bridge, where thermocouples are tack welded for recording temperature signal

For the comparability of results the geometry of the flat solid specimen is equal for all tested materials. Under the condition of passive (unforced) cooling the geometry of the specimen was adapted in order to meet cooling times *t*_8.5_ of <5 s if the maximum temperature during heating reached *T*_max_ = 1150 °C. The narrow region of the specimen having a length of 10 mm and a quadratic cross section of 5.5 mm edge length (Fig. 1).

**Fig. 2:**
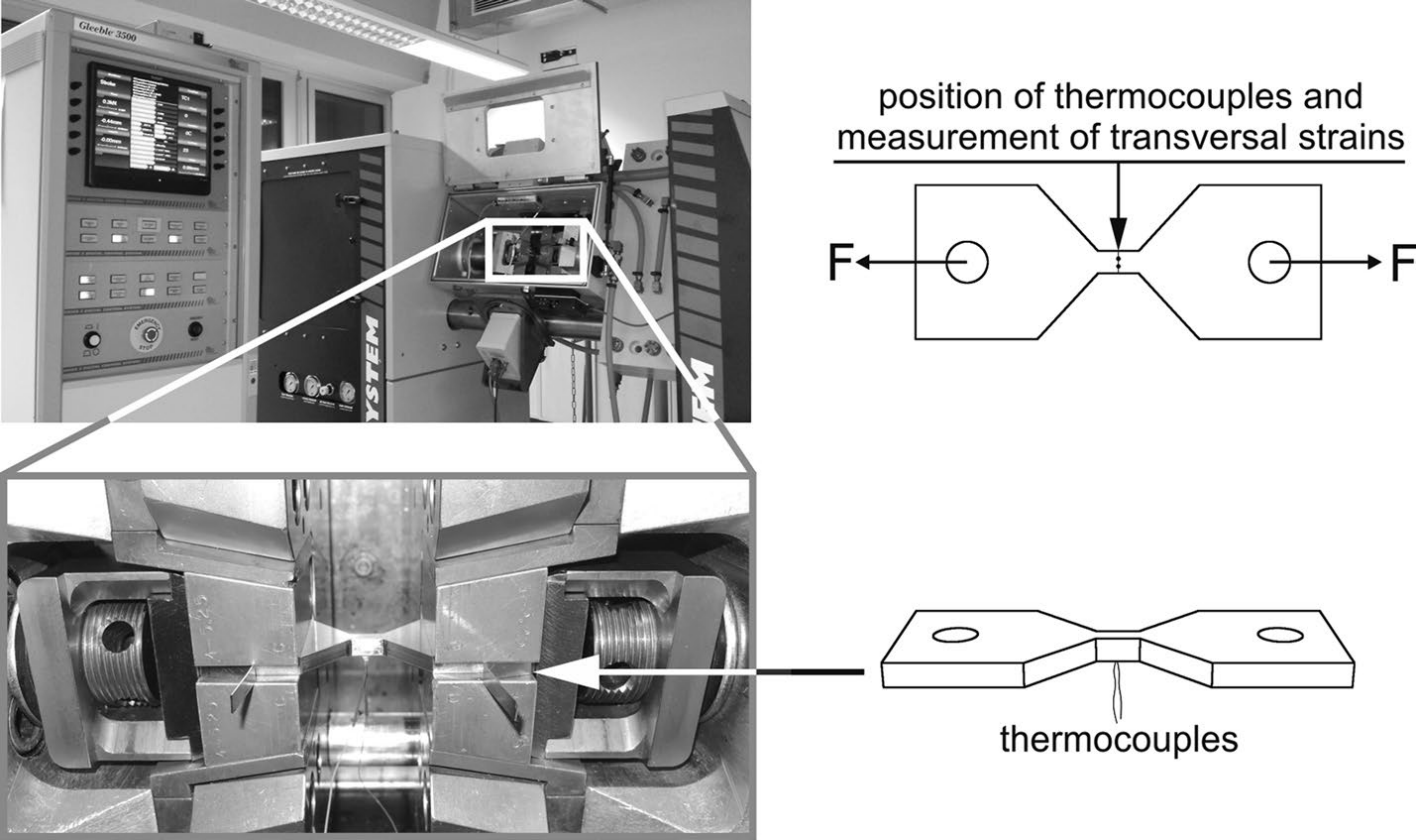
*Gleeble* ^®^ 3500 facility with clamped specimen (IWF—Otto von Guericke Universität). Zoomed part of the measurement chamber, where the specimen (Fig. 1) to be tested is clamped into the two electrically conductive clamping chucks

The TRIP-experiments where realized by use of a *Gleeble*^®^ 3500 facility provided by the *IWF* at the “Otto von Guericke Universität”. In Fig. 2 the *Gleeble*^®^ 3500 facility with zoomed part of the measurement chamber is presented. Now, some characteristic features of *Gleeble*^®^ experiments conducted within this study will be described:

- The specimen to be tested (Fig. 1) is clamped into two electrically conductive clamping chucks by use of fixing bolts connected with the holes of the specimen.
- Heating up of the specimen was done with alternating current, which results by the different electrical potential of the clamping chucks.
- The force application on the specimen happens by force transmission due the fixing bolts inserted in the holes.
- A heated specimen exhibits a symmetric, but non-uniform temperature distribution in axial direction, where the maximum temperature is located in the middle of the flat bridge.
- Thermocouples are tack welded at the middle of the flat bridge, where the maximum temperature is expected.
- Stress to be measured is evaluated by the applied force related to the actual cross-sectional area on the high of the tack welded thermocouples.
- The actual cross-sectional area is determined by use of a C-gauge, which measures the transversal length of the flat bridge at thermocouple position.
- In order to isolate the flat specimens during experiments from outer atmosphere the measurement chamber was filled with argon gas.

### Thermal design

**Fig. 3:**
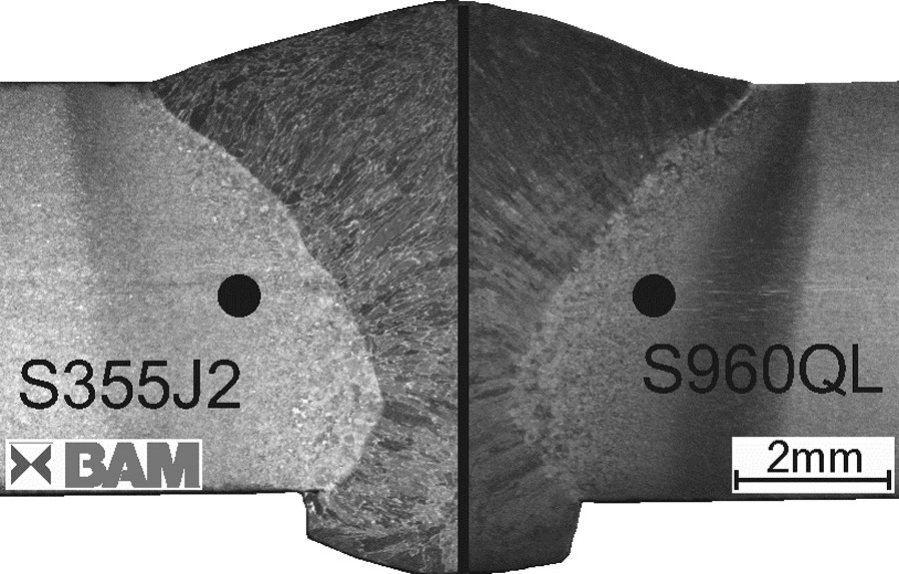
Cross-section. Because of nearly symmetry only one half of the cross-section for GMA welded plates (S355J2+N, S960QL) is shown. *Black dots* represent position of average temperature-peak cycles (within HAZ) found by analyzing the thermal part of a FEA-simulation of the GMA welded plates

In order to provide a material data base for numerical welding simulation, the tested steels (S355J2+N, S960QL) are thermally loaded in accordance to the thermally average HAZ conditions of the GMA welded sheets. Thus, the extent of grain growth and precipitation will be similar like in the HAZ of the welded sheets. Hence, the transient thermal field of the performed GMA weld experiments was reconstructed by a corresponding finite element model, which is exemplarily explained in detail for the GMA weld joint of steel S355J2+N in Neubert et al. (2016). This enables to locate the position of the average thermal history within the volume of the HAZ of the GMA welded plates, which is indicated by the black dots in Fig. 3.

**Fig. 4:**
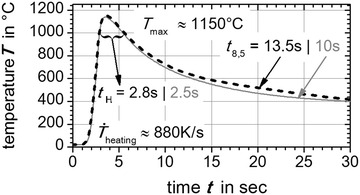
Average temperature-peak cycles for S355J2+N (*black dashed line*) and S960QL (*grey full line*). Characteristics are maximum peak temperature—${\text{T}}_{ \hbox{max} }$, holding time above 1000 °C—${\text{t}}_{\text{H}}$, cooling down time from 800 to 500 °C—*t*_8.5_ and heating up rate—${\dot{\text{T}}}_{\text{heating}}$

**Fig. 5:**
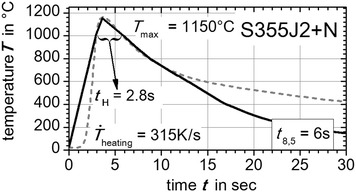
*Gleeble* ^®^ reproduced temperature cycle. With *Gleeble* ^®^ 3500 facility reproduced (*black curve*) average temperature-peak cycle (*grey dashed curve*) for S355J2+N

**Fig. 6:**
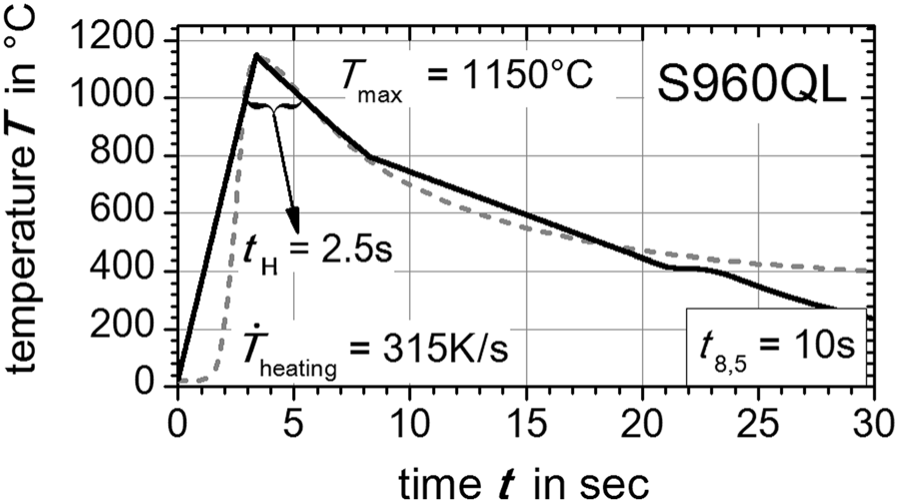
*Gleeble* ^®^ reproduced temperature cycle. With *Gleeble* ^®^ 3500 facility reproduced (*black curve*) average temperature-peak cycle (*grey dashed curve*) for S960QL

**Fig. 7:**
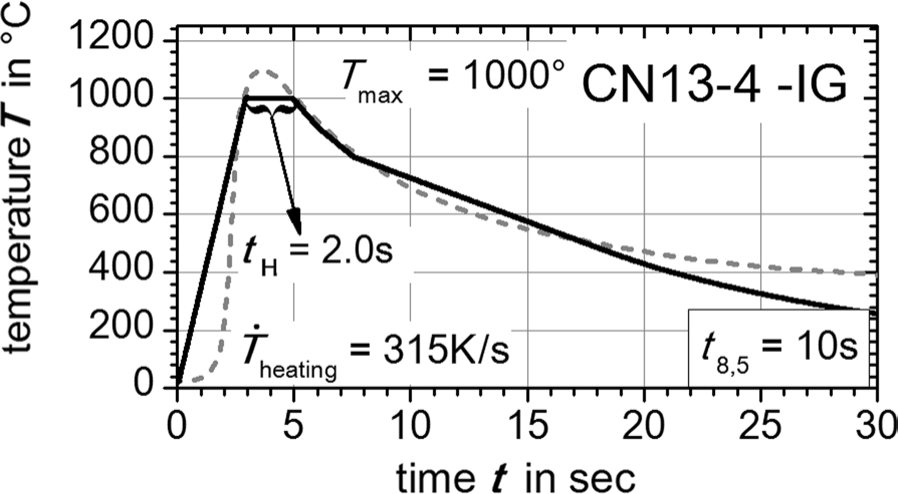
Gleeble^®^ reproduced temperature cycle. With *Gleeble* ^®^ 3500 facility reproduced (*black curve*) measured temperature-peak cycles (*grey dashed curve*) for CN13-4-IG

On this position (black dots in Fig. 3) the thermal cycles were extracted from numerical weld simulation. The extracted numerically calculated thermal cycles are shown in Fig. 4. The resulting characteristics like the heating up rate ${\dot{\text{T}}}_{\text{heating}}$, the holding time ${\text{t}}_{\text{H}}$ above 1000 °C, the maximum peak temperature ${\text{T}}_{ \hbox{max} }$ and temperature evolution above 800 °C were reproduced by the *Gleeble*^®^ facility basing on user defined programming guidelines. Therefore, the middle of the flat bridge of the flat specimen was treated with a thermal cycle exhibiting these characteristics. For the super martensitic filler material CN13-4-IG the same method was applied, but here a measured thermal cycle near the fusion line of the GMA welded plates was directly used to derive thermal weld characteristics (grey dashed curve in Fig. 7). In Figs. 5, 6 and 7 the applied thermal cycles (black curves) rebuilt by the thermo-mechanical simulator *Gleeble*^®^ 3500 are presented. In order to decrease the skin-effect the heating up of the specimen was done with alternating current. Additionally, the heating up rate was reduced to a third for all three materials (S355J2+N, S960QL, CN13-4-IG). Consequently, a better uniformly heated cross-section of the specimen can be obtained. Nevertheless, the heating up rate above 800 °C is modeled well. For S960QL and CN13-4-IG the *t*_8.5_-time of 10 s is well described by the reproduced thermal cycles. However, for S355J2+N a *t*_8.5_-time of 6 s was chosen to shift the solid phase transformation towards lower start temperatures, which results in a production of a nearly pure martensitic solid phase with small amounts of bainite. Apart from that, characteristics like holding time ${\text{t}}_{\text{H}}$ above 1000 °C, maximum temperature ${\text{T}}_{ \hbox{max} }$ and the temperature evolution above 800 °C agree well for the steel samples. The same can be stated for the filler material CN13-4-IG. However, in case of the filler material CN13-4-IG the maximum temperature was set to ${\text{T}}_{ \hbox{max} }$ = 1000 °C to minimize the thermal effects (precipitation and grain grow) since the specimens are taken out of multi pass weld metal test specimens.

### Mechanical design

To ensure that the yield point of the softer austenitic phase is not exceeded already before the beginning of the solid state transformation from γ-phase (austenite) to α-phase (martensite, bainite, ferrite) and having sufficiently high working forces which are applied completely before the beginning of (γ → α)-phase transformation, the critical yield stress must be estimated and the corresponding temperature of (γ → α)-phase transformation has to be known. Hence, before conducting TRIP-experiments some preliminary tests with the *Gleeble*^®^ 3500 facility were performed. In this context, hot (austenitic) tensile tests and dilatometric measurements on all used materials (S355J2+N, S960QL, CN13-4-IG) were executed. For strain rates of $\dot{\varepsilon }$ = 0.05 %/s the yield strength *R*_p0.2_ at *T* = 900 °C was measured and results in *R*_p0.2_ = 65 MPa for S355J2+N, *R*_p0.2_ = 71 MPa for S960QL and *R*_p0.2_ = 90 MPa for CN13-4-IG.

**Fig. 8:**
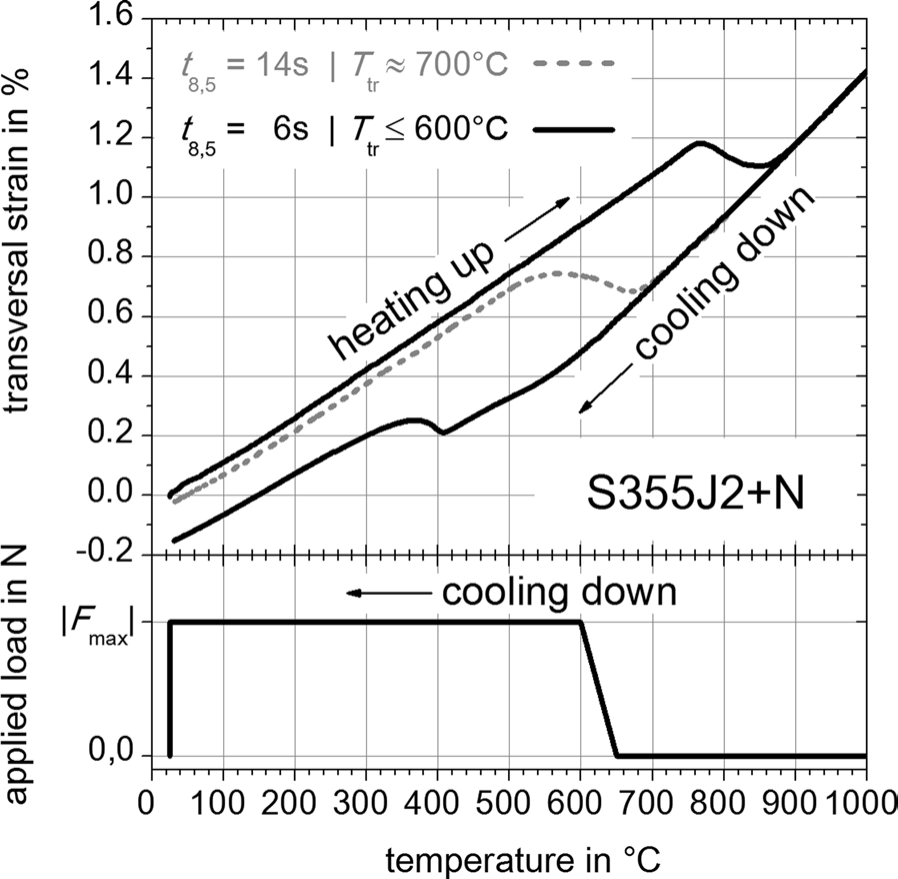
Free dilatometric curve for steel S355J2+N with external load concept. With *Gleeble* ^®^ 3500 facility measured free dilatometric curves. For *t*_8.5_ = 14 s (*grey dashed curve*) the onset temperature of (γ → α)-phase transformation is *T*_tr_ ≈ 700 °C and for *t*_8.5_ = 6 s (*black curve*) results *T*_tr_ ≤ 600 °C

**Fig. 9:**
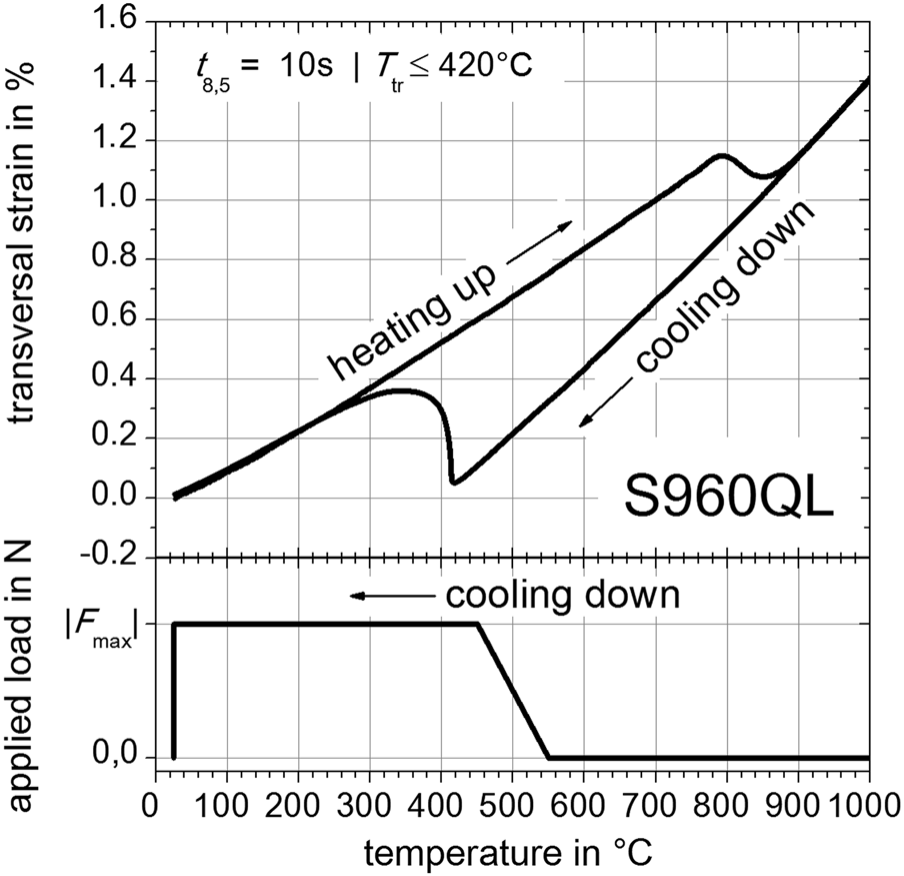
Free dilatometric curve for steel S960QL with external load concept. With *Gleeble* ^®^ 3500 facility measured free dilatometric curves. For *t*_8.5_ = 10 s (*black curve*) the onset temperature of (γ → α)-phase transformation is *T*_tr_ ≤ 420 °C

**Fig. 10:**
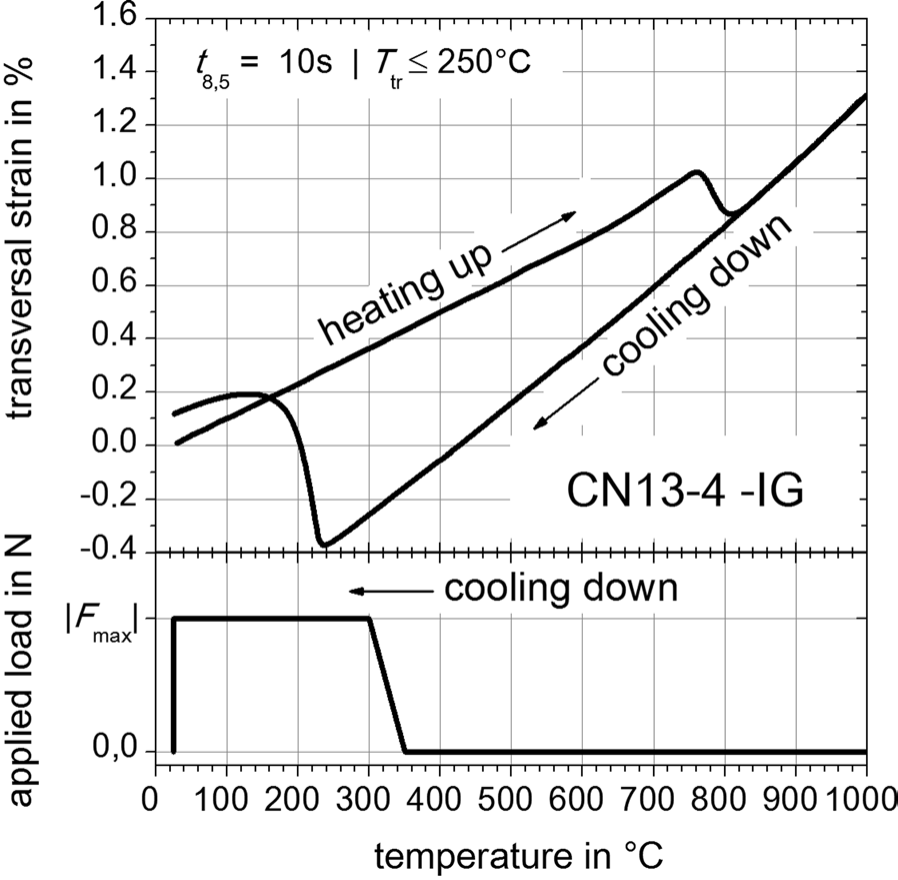
Free dilatometric curve for super martensitic filler fire CN13-4-IG with external load concept. With *Gleeble* ^®^ 3500 facility measured free dilatometric curves. For *t*_8.5_ = 10 s (*black curve*) the onset temperature of (γ → α)-phase transformation is *T*_tr_ ≤ 250 °C

The results of free dilatometric tests (without externally applied loads) are shown in Figs. 8, 9 and 10. For the cooling down time in the range of the average temperature-peak cycles (S355J2+N, Fig. 4) with *t* _8.5_ = 14 s, indicated by the grey dashed dilatometric curve in Fig. 8, the onset temperature of (γ → α)-phase transformation *T*_tr_ is approximately 700 °C. For *t*_8.5_ = 6 s, indicated by the black dilatometric curve in Fig. 8, the temperature *T*_tr_ is less than or equal to 600 °C. Concerning a cooling time of *t*_8.5_ = 6 s the bulk of (γ → α)-phase transformation takes place below 420 °C. Consequently, to improve the measuring reliability of the TRIP-experiments for S355J2+N the cooling time was adapted to *t* _8.5_ = 6 s, which results in a mixture of small amounts of bainitic and nearly pure martensitic solid phases. Concerning S960QL the free dilatometric curve for *t*_8.5_ = 10 s is presented in Fig. 9, which shows that *T*_tr_ is approximately 420 °C. For material CN13-4-IG *T*_tr_ is less than or equal to 250 °C.

Based on these results the temperature dependent increasing of the externally applied loads during cooling can be designed. The resulting temperature dependent external load concept is illustrated in the bottom area of Figs. 8, 9 and 10 and follows the experimental plan given by Table 2. The applied loads *F* are completely imposed between the temperature ranges of 650–600 °C (S355J2+N, Fig. 8), 550–450 °C (S960QL, Fig. 9) and 350 to 300 °C (CN13-4-IG, Fig. 10) during cooling down before reaching *T*_tr_. After reaching room temperature the external applied loads are completely reset. But free dilatometric curves exhibits also remaining plastic strains without the presence of external applied loads. These strains contribute to the TRIP-strains obviously caused by geometry induced internal forces of the flat specimen and caused by prestress due to manufacturing process of the base material and the shaping of the flat specimen.

**Table 2:** Experimental plan for TRIP-experiments

Material	External applied load *F* (N) in |*F*_max_| (two series)					
S355J2+N	+2000	−2000	+1500	−1500	+800	−800
S960QL	+2400	−2400				
CN13-4-IG						
Load case (A, B, C)	≈±66	≈±80	≈±50		≈±26	
	A		B		C	
	Uniaxial stress *σ* (MPa)					

Furthermore, due to the preliminary test the assumption can be drawn, that regarding the experimental plan (Table 2) the yield point of the involved austenitic phases are not exceeded during the TRIP-experiments. Because of scattering in the data the experiments are repeated two times.

## Results and discussion

The main objective of the presented study deals with the experimental determination of the material specific TRIP-parameter K by means of a simplified and thus cost- and time-efficient engineering approach. Considered materials are the mild-strength steel S355J2+N, the high-strength steel S960QL and the super martensitic filler material CN13-4-IG. The results and important consequences of experimental determination of TRIP-parameter K will be presented in the subsequent paragraphs.

### Determination of TRIP-parameter K

**Fig. 11:**
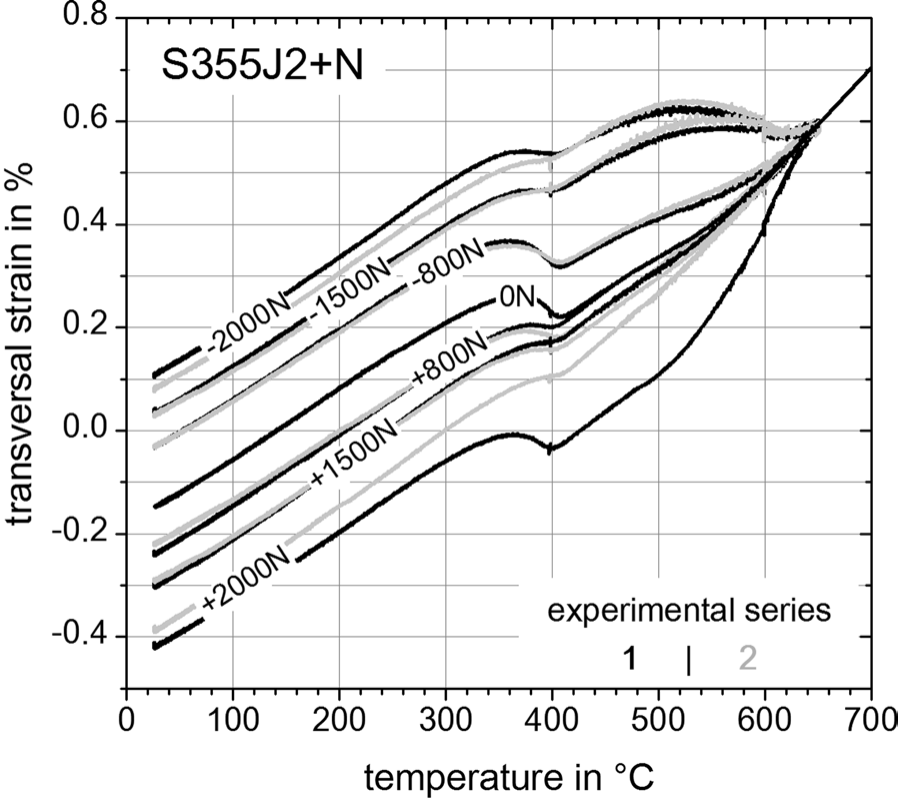
Last parts of dilatometric curves for steel S355J2+N. Last parts of dilatometric curves representing (*γ* → *α*)-transformation for S355J2+N with remaining TRIP-strains at *T* = 25 °C

**Fig. 12:**
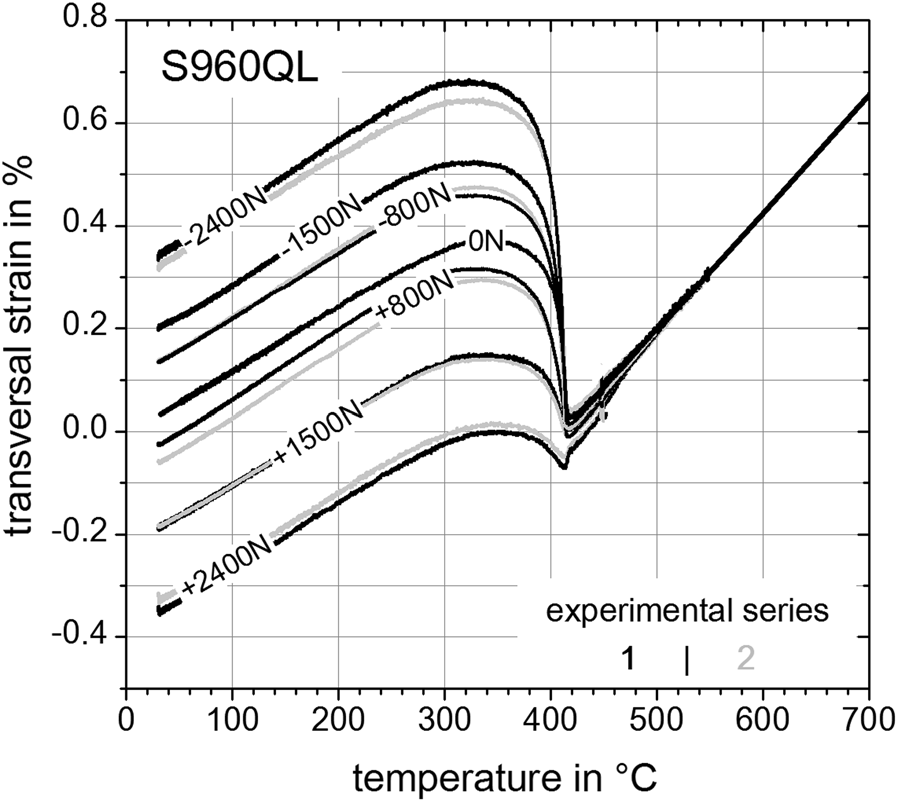
Last parts of dilatometric curves for steel S960QL. Last parts of dilatometric curves representing (*γ* → *α*)-transformation for S960QL with remaining TRIP-strains at *T* = 25 °C

**Fig. 13:**
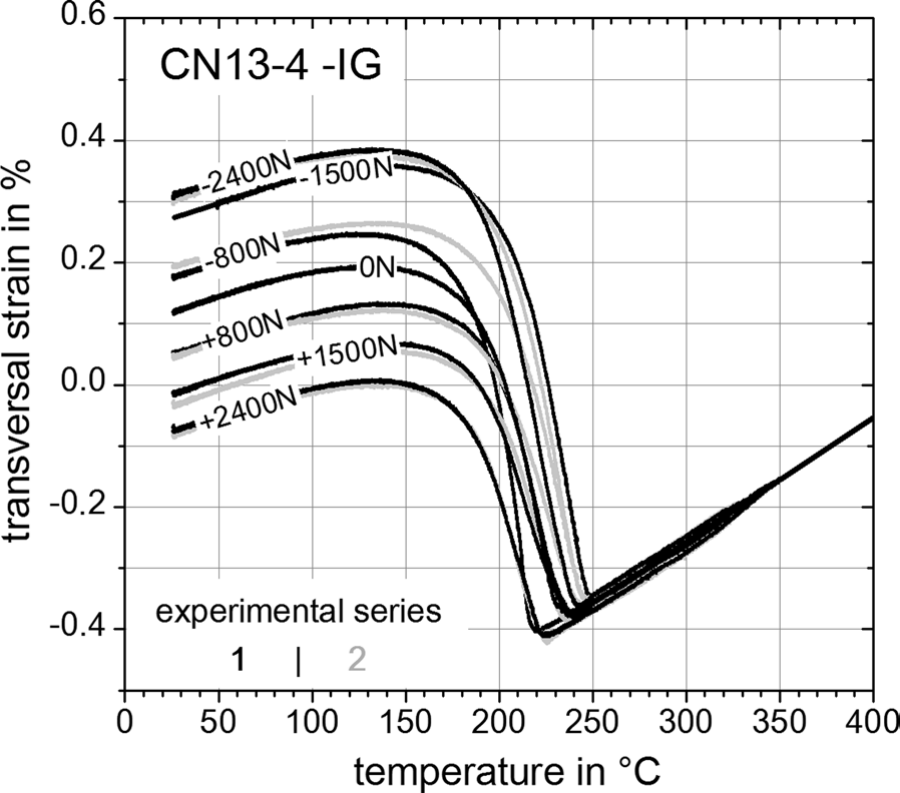
Last parts of dilatometric curves for super martensitic filler wire CN13-4-IG. Last parts representing (*γ* → *α*)-transformation for CN13-4-IG with remaining TRIP-strains at *T* = 25 °C

In this section the results of the *Gleeble*^®^ 3500 experiments comprising the TRIP-strains are presented and evaluated. Figures 11, 12 and 13 show the last parts of the dilatometric curves during cooling down at room temperature with remaining plastic strains after relieving the external applied loads (Table 2). Except for the neutral load cases with *F* = 0 N and for unusable results two measured transversal strains for a given load case exists as indicated by the black and grey curves.

Inferences between the results of Figs. 11, 12 and 13 can be reasoned as follows. Firstly, all materials examined within this study exhibit different transformation behaviour or transformation kinetics, respectively. In case of steel S355J2+N (Fig. 11) a mixture of bainite and martensite is present, where bainite starts to transform at *T*_tr_ ≈ 600 °C and martensite starts to transform at *T*_tr_ ≈ 410 °C. For steel S960QL (Figs. 11, 12) a pure martensitic transformation occurs with a starting temperature at *T*_tr_ ≈ 420 °C. Considering the case of super martensitic filler material CN13-4-IG (Fig. 13) a pure martensitic transformation with an average starting temperature at *T*_tr_ ≈ 250 °C is visible. Secondly, different remaining TRIP-strains with no external applied load (*F* = 0 N) are obvious for each tested material. These remaining TRIP-strains can be caused due to specimen geometry induced internal forces and by prestresses due manufacturing process of the basic material (rolling and quenching process). Also the surface processing to carve out the contour of the flat specimens can introduce prestresses which can cause remaining TRIP-strains with no external load.

Considering the TRIP-experiments of published previous work (Franz et al. 2004; Ossenbrink 2009; Besserdich et al. 1994; Dalgic et al. 2008; Taleb et al. 2001; Besserdich et al. 2000) and following an engineering approach with respect to the magnitude of K the following assumptions and simplifications are made:

1. TRIP-parameter K is independent of
  I involved type of (γ → α)-phase transformation,
  II stress state like tensile or pressure,
  III temperature.
2. Linear behaviour of TRIP-parameter K within the range of performed experiments.
3. $\varepsilon_{\text{tp}} = 2\widetilde{{\varepsilon_{\text{tp}} }}$ with $\varepsilon_{\text{tp}}$—longitudinal and $\widetilde{{\varepsilon_{\text{tp}} }}$—transversal TRIP-strains.
4. TRIP-strains due to specimen geometry induced internal forces and TRIP-strains caused by prestress due to manufacturing process of the flat specimen exhibit every load case with the same size.

This leads to the following evaluation method which considers the formation of a mean value, with $\varepsilon_{\text{tp}}$—longitudinal TRIP-strain (mean value), $\varepsilon$—total transversal strain, *K*—TRIP-parameter, *i*—experimental series (Figs. 11, 12, 13) and *j*—load case (Table 2):

5 $${\text{Mean}}\;{\text{value:}}\;\varepsilon_{\text{tp}} (\sigma )_{j} = \frac{1}{2}\sum\limits_{i = 1}^{2} {\left( { \left| {\varepsilon \left( {\sigma < 0} \right)_{i,j} - \varepsilon \left( {\sigma > 0} \right)_{i,j} } \right|} \right)} ;j\text{ := }\left\{ {A, B, C} \right\}$$

6 $$K = \left. {{\text{d}}\varepsilon_{\text{tp}} (\sigma )/\left| {{\text{d}}\sigma } \right|} \right|_{\sigma \to 0}$$

**Fig. 14:**
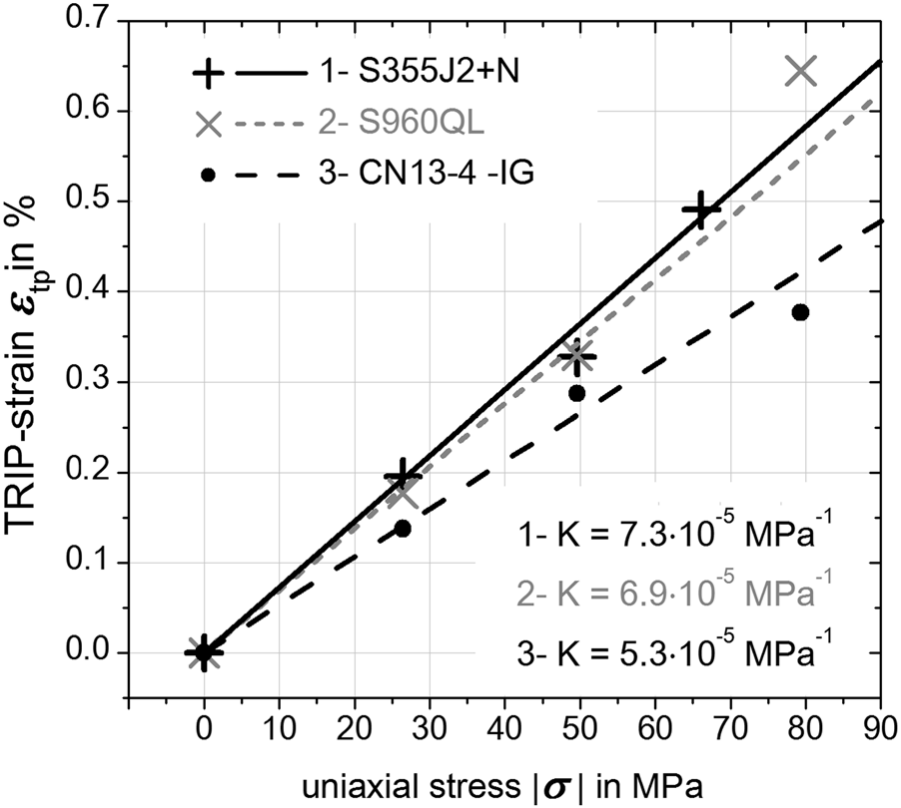
TRIP-strains. With Eq. 5 calculated TRIP-strains for S355J2+N (*grey dashed curve*), S960QL (*black curve*), CN13-4-IG (*black dashed and dotted curve*) and with Eq. 6 calculated TRIP-parameter *K*

Using Eq. 5 together with the aforementioned assumptions (2) and (4) the TRIP-strains due to internal thermal forces or prestresses are eliminated. This is an important issue in contrast to other approaches in literature because now with no external applied load no TRIP-strain is induced and consequently the mean value $\varepsilon_{\text{tp}}$ can be set to zero for *σ* = 0. All other calculated TRIP-strains are shown in Fig. 14. With Eq. 6 for evaluating the slope at *σ* → 0 leads to the TRIP-parameters K = 7.3 × 10^−5^ MPa^−1^ for S355J2+N, K = 6.9 × 10^−5^ MPa^−1^ for S960QL and *K* = 5.3 × 10^−5^ MPa^−1^ for CN13-4-IG. Considering TRIP-results for steels with a carbon content ≤0.2 wt% (Franz et al. 2004) a K-value for material “20NiCrMo4-3-5” with K_1_ = 7.2 × 10^−5^ MPa^−1^ can be found in literature. The analyzed heat of S355J2+N had a carbon content of 0.14 wt% which results in a TRIP-parameter of K_2_ = 7.3 × 10^−5^ MPa^−1^ (Fig. 14). Following the investigations presented in (Ossenbrink 2009) for steel S355J2G3 a TRIP-parameter of K_3_ = 9.6 × 10^−5^ MPa^−1^ was determined. For the cases K_1_ and K_3_ a complex and thus costly hollow cylindrical specimen geometry was used. It can be concluded that K_1_ < K_2_ < K_3_ and as well as all TRIP-parameters have the same magnitude.

It is worth noticing that the suitability of the proposed cost-saving flat specimen geometry (Fig. 1) used for the TRIP-experiments and the efficient engineering experimental and evaluation method for determining the TRIP-parameter K could be demonstrated well.

Because of the chemical similarity between the analyzed steels S355J2+N and S960QL, the same experimental and evaluation approach and the similar TRIP-behavior (Fig. 14) the K-result for S960QL with K = 6.9 × 10^−5^ MPa^−1^ can be considered credible regarding the magnitude of K.

In Kasuya et al. (2014) a high-alloy weld metal with low temperature transformation behavior was thermo-physically tested which result in a TRIP-parameter of K = 5 × 10^−4^ MPa^−1^. The typical chemical composition of the tested specimen was 0.04 wt% carbon, 14.3 wt% of chromium and 6.4 wt% of nickel. However, it has to be stressed that in this study the chemically similar high-alloy super martensitic weld metal CN13-4-IG gives a value of K = 5.3 × 10^−5^ MPa^−1^ for the TRIP-parameter (Fig. 14). The discrepancy between the high-alloy weld metals with reduced martensite start temperature is nearly one magnitude. Unfortunately, there was only one single result of a TRIP-tested specimen presented in Kasuya et al. (2014) and the contributions of internal forces to the TRIP-strains has not been compensated. Therefore, a direct comparison of the two different results can hardly be carried out. On the other hand, in addition to the chemical composition that is one influencing factor of the martensite start temperature further parameters like primary austenite grain size (heating rate, peak temperature, holding time) have to be taken into account in order to compare different thermos-physical simulations.

Due to the evaluation method that concerns the mean value between pressure and tensile external load values (Eq. 5) the TRIP-strains can be evaluated for the given geometry. Furthermore, the influence of inner stresses due the delivery condition of the base material or mechanical fabrication process of the specimens on the TRIP-strains, are compensated by this evaluation method.

## Conclusions

In this study the TRIP-parameters K for the low-alloyed steels S355J2+N and S960QL and for the super martensitic filler material CN13-4-IG were experimentally determined by use of a *Gleeble*^®^ 3500 facility. Based on the obtained results the following conclusions have been drawn:

1. Within the *Gleeble*^®^ experiments a flat specimen geometry in combination with a reasonable evaluation method was successfully applied to determine the TRIP-parameter K for the three examined materials.
2. Evaluating experiments the transformation plasticity was assumed to be independent from temperature, present solid phases and stress state like tensile or pressure.
3. The determined TRIP-parameter K for the steel S355J2+N with K_S355J2+N_ = 7.3 × 10^−5^ MPa^−1^ corresponds to literature values. Also the determined TRIP-parameter K for the chemically similar steel S960QL with K_S960QL_ = 6.9 × 10^−5^ MPa^−1^ seems to be in reasonable accordance. Furthermore, the TRIP-parameter K for the super martensitic filler material CN13-4-IG was determined to K_CN13-4_-IG = 5.3 × 10^−5^ MPa^−1^, which is one order of magnitude smaller compered to published literature values of a chemically similar material.
4. Compared to conventional approaches, the presented method within this study enables the experimenter to determine the TRIP-parameter K in a cost- and time saving way.

## Methods

- Thermomechanical simulation by use of a *Gleeble*^®^ 3500 facility
- *Gleeble*-experiments were designed by numerically calculated thermal cycles of a real gas metal arc weld
- Use of a simplified flat specimen for *Gleeble*^**®**^ experiments to determine the transformation induced plasticity for two steels and one welded filler fire
- Application of a simplified evaluation method to evaluate the experimental results of *Gleeble*^®^ experiments
- Validation of the experimental approach by literature results concerning the transformation induced plasticity parameter K
- Objects of study were the low-alloyed mid-strength steel S355J2+N , the low-alloyed high-strength steel S960QL and the welded super martensitic (high-alloyed) filler wire CN13-4-IG.

## Abbreviations

*F*: force
*i*: experimental series
*j*: load case
*K*: TRIP-parameter
*R*_p0.2_: yield strength at plastic strain equal to 0.2 %
*T*: temperature
*Ṫ*: heating up rate
*T*_max_: maximum temperature
*T*_tr_: onset temperature of phase transformation
*t*: time
*t*_8.5_: time for cooling down from 800 to 500 °C
*t*_H_: holding time above 1000 °C
*w*: fraction of transformed material
*α*: designation of ferritic/bainitic/martensitic phase
*γ*: designation of austenitic phase
*ɛ*: total strain
$\dot{\varepsilon }$: time dependent total strain rate
$$\varepsilon_{\text{el}}$$: elastic strain
$$\varepsilon_{\text{pl}}$$: plastic strain
$$\varepsilon_{\text{th}}$$: thermal strain
$\varepsilon_{\text{tp}}$: longitudinal transformation induced plasticity strain
$\widetilde{{\varepsilon_{\text{tp}} }}$: transversal transformation induced plasticity strain
$$\varepsilon_{\text{tr}}$$: transformation strain
*σ*: uniaxial macro stress
